# Objective and Subjective Assessments of Sleep in Children: Comparison of Actigraphy, Sleep Diary Completed by Children and Parents’ Estimation

**DOI:** 10.3389/fpsyt.2020.00495

**Published:** 2020-06-10

**Authors:** Stéphanie Mazza, Hélène Bastuji, Amandine E. Rey

**Affiliations:** ^1^HESPER Laboratory, Université Lyon 1, Université de Lyon, Lyon, France; ^2^Central Integration of Pain, Lyon Neuroscience Research Center, Inserm U1028, Université Lyon 1, Université de Lyon, Lyon, France; ^3^Unité d’Hypnologie, Service de Neurologie Fonctionnelle et d’Épileptologie, Hôpital Neurologique, Hospices Civils de Lyon, Bron, France

**Keywords:** sleep diary, actigraphy, parents’ report, school-based children, sleep measurements

## Abstract

In research and clinical contexts, parents’ report and sleep diary filled in by parents are often used to characterize sleep-wake rhythms in children. The current study aimed to investigate children self-perception of their sleep, by comparing sleep diaries filled in by themselves, actigraphic sleep recordings, and parental subjective estimation. Eighty children aged 8–9 years wore actigraph wristwatches and completed sleep diaries for 7 days, while their parents completed a sleep-schedule questionnaire about their child’ sleep. The level of agreement and correlation between sleep parameters derived from these three methods were measured. Sleep parameters were considered for the whole week and school days and weekends separately and a comparison between children with high and low sleep efficiency was carried out. Compared to actigraphy, children overestimated their sleep duration by 92 min and demonstrated significant difficulty to assess the amount of time they spent awake during the night. The estimations were better in children with high sleep efficiency compared to those with low sleep efficiency. Parents estimated that their children went to bed 36 min earlier and obtained 36.5 min more sleep than objective estimations with actigraphy. Children and parents’ accuracy to estimate sleep parameters was different during school days and weekends, supporting the importance of analyzing separately school days and weekends when measuring sleep in children. Actigraphy and sleep diaries showed good agreement for bedtime and wake-up time, but not for SOL and WASO. A satisfactory agreement for TST was observed during school days only, but not during weekends. Even if parents provided more accurate sleep estimation than children, parents’ report, and actigraphic data were weakly correlated and levels of agreement were insufficient. These results suggested that sleep diary completed by children provides interesting measures of self-perception, while actigraphy may provide additional information about nocturnal wake times. Sleep diary associated with actigraphy could be an interesting tool to evaluate parameters that could contribute to adjust subjective perception to objective sleep values.

## Introduction

Twenty-five to 40% of healthy children and adolescents suffer from behavioral sleep problems affecting quality, timing or duration (1, 2). Insufficient quantity or quality of sleep predicts the development of health issues (3, 4), cognitive impairment and behavioral problems (5). Thus, the assessment of sleep-wake rhythms is valuable for the identification and management of sleep difficulties.

A variety of objective and subjective tools have been used to assess sleep-wake rhythms in children and adolescents, including polysomnography (PSG), actigraphy, sleep diary, and parental report questionnaires. These methods differ with respect to cost, duration, ease of use, level of intrusiveness, and type of data they provide. Laboratory-based PSG is deemed the “gold standard” for measuring objectively sleep parameters and architecture or for establishing the presence and severity of sleep disorders in children such as obstructive sleep apnea. However, PSG is a relatively expensive procedure that provides information about sleep for usually one or 2 nights, most of the time in an unfamiliar environment that can make PSG challenging and even frightening for children (6). Actigraphy is a non-invasive method to assess sleep-wake rhythms in the child’s natural setting for extended periods of time, with a reasonable validity and reliability compared to PSG (7) or videosomnography (8). Actigraph is a wristwatch-like device containing an accelerometer providing a continuous monitoring of motor activity. This activity is translated to epochs of wake (activity) or sleep (inactivity) using a device-specific algorithm. Actigraphy recordings are often completed by a sleep diary filled by children’s parents. Subjective tools, such as sleep diary and questionnaires, require minimum supervision and provide information related to personal perception of sleep. Sleep diary is a useful methodology to record information on sleep on a night-by-night basis (e.g., bedtime, sleep duration, sleep onset latency, night awakenings) and reflects a subjective global appraisal of each night sleep. Parental reports with questionnaires have often been used to evaluate children’ sleep (9). This method can provide a detailed description of the child’s sleep schedule, night awakenings and sleep-related behaviors such as bedtime resistance, parasomnia (e.g., sleepwalking and night terrors) and markers of sleep-disordered breathing (snoring, restless and disrupted sleep). Although simple questionnaires are suitable for screening and monitoring of a large population, sleep diaries are preferred for more detailed assessment of sleep-wake rhythms.

Beyond psychological factors (for instance depressive disorders), sleep quality itself influences the congruence between subjective and objective measures (10). Whereas good sleepers showed a more suitable perception of their sleep duration, the accuracy of patients with sleep-disorders varied widely (11, 12). Studies comparing objective and subjective assessments in large cohorts have found that adults overestimated their mean habitual sleep time by approximately 1-h when using sleep diary compared to PSG recording (13) or with questionnaires compared to actigraphy (14). In most studies investigating sleep in children, sleep diaries are filled in by parents. When parents have to estimate the sleep habits of their child, it has been shown that they tended to estimate with more accuracy sleep schedule variables than time awake in bed (sleep latency and night awakenings) (15). Moreover, the consistency of their reports decreased when the monitoring lasted a long time (16). Specifically, parents tend to report earlier bedtimes and later wake-up times in comparison with actigraphic measures, which average overestimation of sleep duration ranging from 30 to 113 min per night in different studies (17, 18). In adolescents, sleep parameters estimated with sleep diary and actigraphy measures are positively correlated especially during school days (19). They overestimated their sleep duration by approximately 1 h compared to actigraphy and underestimated their night awakenings (20). Short et al. (17) showed that between 13 to 17 years old, adolescents reported more accuracy than parents’ reports; the latter overestimated sleep and underestimated bedtimes, suggesting that the use of adolescent reports should be preferred. There are few studies comparing sleep diary filled in by children and actigraphy. Most of them studied pre-adolescents or adolescents (17, 20–23). However, research on cognitive development, psychometric studies and longitudinal research indicate that children, as young as 8 years of age, can successfully provide valuable information about their own health when appropriate assessment methods are applied (24).

To our knowledge, this is the first study to investigate children’s self-perception of their sleep, by comparing sleep diaries filled in by nonclinical children aged between 8 and 9, actigraphic sleep recordings, and parental subjective estimation during 7 days in a non-clinical population. Analyses were performed for the whole week and school days and weekends separately. To assess potential differences in sleep perception triggered by sleep quality, we compared children with high or low efficiency. We also evaluated the level of agreement and correlation between sleep parameters derived from these three measures.

## Methods

### Participants

This study was part of a larger investigation examining the effects of a sleep education program on sleep, cognitive and academic performances in children. One hundred and thirty school-age children aged 8 to 9 years were recruited in five elementary schools. In the present study, the sample included the 100 children from the four schools where actigraphic measures were performed. Participants were instructed to wear an actigraph device, nights and days, during 7 consecutive days, and to complete a sleep diary each morning at school. Their parents had to fulfil a self-constructed Sleep-Schedule Time Questionnaire during the same period (see materials). The data analyzed for the current study corresponds to the baseline period of the larger study (before the sleep education program) and was collected during the fall to minimize potential seasonal effects. The protocol has been approved by the local Ethics Committee (#IRB00010290-2016-08-03). Written informed consent was obtained from the parents and assent was obtained from the children prior to participation.

All participants had no history of psychiatric or neurological illness, developmental disorder or learning disability, according to parental reports. Sleep disorders were assessed using the Sleep Disturbance Scale for Children (25). Four children reached a pathological sub-score on the scale and were excluded from the analyses.

As five or more usable night recordings are recommended to obtain reliable measures of sleep for actigraphy in children (26), analyses were performed on actigraphic data with at least six usable nights including a minimum of one night during weekends (16% of the sample had only one night during weekend). Sleep diary and actigraphic recording were incomplete in 11 children and parents’ report was missing for 9 children. Thus, a total 76 children (43 girls) aged 8–9 years (*M* = 8.5 years, *SD* = 0.3) with a complete set of data (sleep diary, actigraphic recording and parents’ report) were included for analyses. Additional sample characteristics are presented in **Table 1**.

**Table 1:** Sample characteristics.

Variable	*N* or %
Age of children, mean (SD) [range]	8.5 (0.3) [7.9–9.3]
Sex, percentage F M	56.6 43.4
Number of children per family, mean (SD) [range] Of which first born, percentage	2.3 (0.7) [1–4] 31
Sleep Disturbance Scale in Children, mean (SD) [range] Insomnia Parasomnia Sleep related breathing disorders Circadian rhythms sleep-wake disorders Central disorders of hypersomnolence	12.8 (4.1) [0–20] 11.0 (3.3) [0–17] 7.3 (2.2) [0–11] 5.7 (2.2) [0–11] 3.3 (0.7) [0–5]
Birth order of child, mean (SD) [range]	1.7 (0.7) [1–3]
Number of electronic media devices at home (television, computers, consoles, tablets), mean (SD) [range]	3.8 (1.5) [1–8]
Children with their own cell phone, percentage	3.9
Children with an electronic media device in their bedroom, percentage No device Consoles Television Computers	85.6 11.8 2.6 0.0

### Measure

#### Objective Sleep Assessment: Actigraphy

Each child was invited to wear an actigraph (Actiwatch 2, Philips Respironics, Bend, OR, USA) on the non-dominant wrist for 1 week (5 school days and 2 weekends). This electronic wristwatch-like accelerometer measures the amount, duration and intensity of movements in free-living settings. Activity counts from the device were collected in 30-s sampling epochs and reflected the peak of acceleration detected and were used to determine sleep and wake intervals. Whether a particular epoch was scored as wake was determined by comparing activity counts for the epoch in question and those immediately surrounding it, to a threshold value (sensitivity threshold). If the total activity count was above the sensitivity threshold, the epoch was scored as wake, and if the total activity count was equal to or below the sensitivity threshold, the epoch was scored as sleep. For this study the sensitivity threshold was set to 40 counts by epoch (medium sensitivity) since this setting has been found to yield the least overestimation or underestimation of sleep or wakefulness for total sleep time and wake after sleep onset compared to PSG in children aged 9–11 years (27) and 6–12 years (28). The Actiwatch 2 devices are equipped with an event marker and a light sensor. At night, when children were in bed ready to fall asleep, they were told to use the event marker to designate their “bedtime”. In the morning, when they woke up, they were instructed to mark the actigraph again. Sleep interval was marked manually for each record on the basis of event marker, activity, and light information. Sleep diary was used to know the timing of device removal. Actigraphic sleep data were analyzed in 30-sec epochs using Actiware Sleep software 6.0.9.

Sleep start and sleep end were determined automatically as the first and last 10 min period respectively in which no more than one epoch was scored as mobile. Automatic analyses were run to extract the following sleep parameters: (a) bedtime (clock time attempted to fall asleep), (b) wake-up time, (c) Total Sleep Time (TST - estimated amount of time scored as sleep, according to the Actiware-Sleep Algorithm), (d) Sleep Onset Latency (SOL - amount of time elapsing from bedtime to the first period of sleep), (e) Wake After Sleep Onset (WASO - number of minutes scored as wake-up time during sleep period). Analyses were performed on actigraphic data when at least six nights including at least one night during weekends were available.

#### Children Self-Report Assessment: Sleep Diary

During 7 consecutive days, children completed a sleep diary each morning at 08:30 am. Instruction for filling in the diary was learned at school and children completed them under the teacher supervision during school days and alone during weekend. The sleep diary consisted in a daily record of sleep parameters. Each 24-h period was represented by a continuous line divided in boxes, one box corresponding to 1 h. Children were told to write the current date, then draw a down arrow to indicate their bedtime and a up arrow to indicate their wake-up time. Then, they shaded in the boxes corresponding to their assumed sleep period and leave boxes unshaded to show wake period during the day or the night. Children were asked to indicate, for each half hour, whether they were awake or asleep. The following parameters were extracted from the sleep diary: (a) bedtime (defined by the down arrow), (b) wake-up time (defined by the up arrow) (c) TST (colored part between time to bed and wake-up time, (d) SOL (uncolored part between time to bed and the beginning of TST), (e) WASO (uncolored part throughout the beginning and the end of TST), and (g) sleep quality, ease of waking and sleepiness scores.

#### Parents’ Report: Sleep-Schedule Time Questionnaire

Bedtime, wake-up time and assumed TST were obtained through a self-constructed questionnaire. The questions were phrased as follows: “Last night, your child went to bed at _____” (bedtime), “This morning, you child woke up at _____” (wake-up time) and “Indicate the total sleep duration of your child last night: _____ (hours and minutes)” (total sleep time). Example entries of 8:00 pm, 06:00 am and 9 h 30 min were given on first row. No answer categories were presented for any question, and information was collected separately for school days and weekends. Sleep onset latency and time awake during the night were not obtained.

### Statistical Analyses

All actigraphy and sleep diary data were visually reviewed by 2 trained assistants. To ensure a good agreement between the two raters, they were first trained by scoring 5 records collectively, then they individually scored 10 identical records to allow discussion of discrepancies. To assess the agreement of the two raters’ observations, a Kendall coefficient was calculated for the 10 identical records. The overall agreement rate was 87%. Statistical analyses were performed using RStudio version 3.2.2 (29) and JAMOVI version 1.0 (30). Normality of distribution was tested using the Shapiro-Wilk test.

#### Sleep Parameters Analysis

Repeated measures ANOVAs were conducted on each sleep parameters with Measures (actigraphy vs. sleep diary vs. parents’ report) as between-subjects factor and Study period (school days vs. weekends) as within-subjects factor. School days refer to Sunday to Friday nights and weekends refer to Friday and Saturday nights. The Greenhouse-Geisser correction was applied when the assumption of sphericity was violated. P values were adjusted for multiple comparison tests through the Bonferroni correction. Values are given as mean ± SDs. The mean difference was calculated between actigraphic and subjective measures (sleep diary and questionnaire) with a 95% confidence interval. Effect size was estimated by using eta-squared or Cohen’s d and was interpreted as small (η² ≤ 0.01, *d* ≤ 0.3), moderate (η² ≤ 0.08, *d* = 0.5), or large (η² ≤ 0.25, *d* = 0.8). The level of significance was set at α < 0.05. To compare children with high and low sleep efficiency, the sample was divided according to the sleep efficiency calculated with actigraphy (ratio of TST divided by TIB for all days) using the median-split criterion and two-tailed student t-test were conducted between the two groups.

#### Sleep Parameters Agreements Analyses

Pearson correlations were performed to assess the potential extent of the association between sleep parameters for (1) actigraphy and sleep diary and (2) actigraphy and parents’ report and (3) sleep diary and parents’ report. Correlation coefficients are sometimes inadequate and can be misleading when assessing agreement between measurements, because they evaluate only the linear association of two sets of observations (a strong correlation does not imply that a good agreement exists between them). Bland Altman plots is a graphical approach to quantify agreement between two quantitative measurements by constructing limits of agreement (31). These statistical limits are calculated by using the mean and the standard deviation of the differences between two measurements. For good agreement, it is recommended that 95% of the data points should lie within +/- two standard deviations of the mean difference (32).

The difference for each subject between (1) actigraphy and sleep diary, (2) actigraphy and parents’ report, and (3) sleep diary and parents’ report for all days, school days and weekends were plotted against their averages. A reference line equal to zero represents perfect agreement between the two measures. The mean bias represents the mean difference between the two measures and limits of agreement are defined as a deviation from the mean to two standard deviations.

A classical test of variance for paired samples based on the bivariate normal distribution that compares the variance of the difference with the variance of the average was calculated according to the Pitman’s test. A *p*-value > .05 suggested a significant difference in the variability between measurements.

## Results

### Comparison of Sleep Parameters Assessed by the Different Measures

Mean (SD) of bedtime, wake-up time, total sleep time, sleep onset latency, and wake after sleep onset assessed by the 3 different measures are presented in **Table 2**.

**Table 2:** Mean, SD and range for each sleep parameters according to actigraphy, sleep diary or parents’ report during all days (the combination of school days and weekends), school days, and weekends.

	Actigraphy Mean (SD) [range] (*N* = 76)	Sleep diary Mean (SD) [range] (*N* = 76)	Parents’ report Mean (SD) [range] (*N* = 76)
**All days**			
Bedtime (hrs:min)	21:44 (00:41) [20:35; 00:16]	21:02 (00:44) [20:00; 23:15]	21:05 (00:25) [20:00; 22:00]
Wake-up time (hrs:min)	07:40 (00:30) [06:05; 08:59]	07:23 (00:37) [06:00; 09:00]	07:48 (00:26) [07:00; 09:30]
TST (min)	528.3 (30.04 [411.6; 625.4]	620.8 (45.6) [498.8; 705.0]	564.7 (36.3) [390.0; 600.0]
SOL (min)	14.7 (11.24 [1.7; 47.1]	7.4 (18.6) [0.0; 92.5]	NA
WASO (min)	33.9 (10.5) [7.4; 62.3]	7.9 (19.0) [0.0; 105.0]	NA
**School days**			
Bedtime (hrs:min)	21:11 (00:40) [20:01; 23:28]	20:43 (00:39) [20:00; 23:00]	20:37 (00:31) [19:30; 22:00]
Wake-up time (hrs:min)	07:13 (00:24) [05:59; 08:08]	07:05 (00:28) [06:00: 08:15]	07:18 (00:29) [07:00; 09:30]
TST (min)	517.6 (45.2) [344.3; 655.3]	622.1 (42.3) [480.0; 693.8]	561.1 (43.4) [390.0; 600.0]
SOL (min)	13.4 (11.2) [0.3; 45.6)	8.9 (23.0) [0.0; 135.0]	NA
WASO (min)	33.6 (11.1) [8.0; 67.1]	7.9 (19.1) [0.0; 120.0]	NA
**Weekends**			
Bedtime (hrs:min)	22:17 (00:56) [20:53; 01:03]	21:22 (01:04) [20:00; 00:30]	21:36 (00:30) [20:30; 22:00]
Wake-up time (hrs:min)	08:06 (00:47) [06:12; 09:55]	07:42 (00:56 [06:00; 10:00]	08:20 (00:37) [07:00; 09:30]
TST (min)	537.9 (30.7) [437.7; 602.5]	619.4 (63.6) [420.0; 750.0]	568.3 (37.8) [390.0; 600.0]
SOL (min)	15.9 (15.2) [0.0; 62.8]	5.9 (17.2) [0.0; 90.0]	NA
WASO (min)	34.4 (13.8) [6.8; 76.5]	7.8 (22.5) [0.0; 200.0]	NA

TST, Total Sleep Time; SOL, Sleep Onset Latency; WASO, Wake After Sleep Onset; NA, Not Available.

ANOVA performed on bedtime showed significant main effects of Measures (*F*_1,75_ = 62.8, *p* < .001, η² = .11) and Study period (*F*_1,75_ = 85.7, p < .001, η² = .38), as well as a significant interaction (*F*_1,75_ = 5.9, *p* = 004, η² = .01). Bedtimes reported in sleep diary and parents’ report significantly differed from actigraphy (all *p*s < .001, cohen’s *d* > .74). Both children’s and parents’ estimations indicated significantly earlier bedtime compared to actigraphy (40.5 and 36.6 min, respectively; 95% CI are presented in **Table 2**). This misperception was found both during school days and weekends (all *p* values < .001, all d values > 50). A significant difference was found during weekends between sleep diaries and parents’ reports, bedtime estimated in sleep diaries was 14.1 min earlier compared to parents’ reports (*t*_75_ = 32.5, *p* = .04, *d* = 0.21) and therefore more distant from actigraphy measure.

When considering wake-up time, a significant main effect of Measures (*F*_1,75_ = 27.2, *p* < .001, η² = .05), Study period (*F*_1,75_ = 101.0, *p* < .001, η² = .42), and a significant interaction (*F*_1,75_ = 7.7, *p* < .001, η² = .01) were observed. Children reported in their sleep diary a wake-up time 16.6 min earlier than measured by actigraphy when considering all days (*t*_71_ = 4.3; *p* < .001, *d* = .37), whereas parents estimated wake-up time 9.7 min later than actigraphy measures (*t*_71_ = -2.7; *p* = .02, *d* = -.23). No significant difference was observed between the three measures during school days (all *p*s > .25). During weekends, wake-up time significantly differed between children’s sleep diary and parents’ reports (-39.6 min, *t*_71_ = 7.9, *p* < .001, *d* = .66). Children estimated waking up 24.3 min earlier compared to actigraphy measures during weekends (*t*_71_ = 4.9, *p* < .001, *d* = .42). Conversely, parents’ estimates were delayed by 16.1 min compared to actigraphy measures (*t*_71_ = -2.9, *p* = .02, *d* = -.24).

There was a significant difference of TST for Measures (*F*_1,71_ = 175.4, *p* < .001, η² = .42), but no significant difference for Study period *(p* = .12). A trend to significant interaction Measures x Study period was also found (*F*_1,71_ = 2.5, *p* = .08, η² = .01). When considering all days, TST was overestimated by 92.4 min in sleep diary (*t*_75_ = -17.1, *p* < .001, *d* = -.1.5) and by 36.5 min in parents’ report (*t*_75_ = -8.6, *p* < .001, *d* =-.73) compared to actigraphy. The difference between children’s and parents’ estimates was 56.0 min (*t*_75_ = -10.7, *p* < .001, *d* = -.91). Whereas actigraphy showed that TST significantly increased during weekends compared to school days (20.3 min, *t*_71_ = -2.6, *p* = .03, *d* = -.22), such difference was not observed in sleep diaries and parents’ estimates (all *p*s = 1.0).

Comparison for WASO and SOL were conducted between actigraphy and sleep diary. Children significantly underestimated their WASO in sleep diaries by more than 25 min (*F*_1,71_ = 163.4, *p* < .001, η² = .37), whatever the Study period (school days: *t*_75_ = 8.9, *p* < .001, *d* = .74; weekends: *t*_75_ = 9.2, *p* < .001, *d* = .77). A significant effect of Measures (*F*_1,71_ = 14.8, *p* < .001, η² = .04) but no effect of Study Period (*p* = .87) nor interaction (*p* = .16) were observed for SOL. SOL estimated in sleep diary was 10.0 min smaller than SOL estimated with actigraphy during week-end (*t*_75_ = 3.1, *p* = .002, *d* = .31).

### Sleep Estimation in “Poor” Versus “Good” Sleepers

Children were categorized as high efficiency sleepers (60.5% of girls) and low efficiency sleepers (52.6% of girls) based on a median split on the all days sleep efficiency measured with actigraphy and defined as 89.2% (low efficiency sleepers: 86.0 ± 3.3% vs high efficiency sleepers: 91.5 ± 1.6%, *p* < .001, *d* = 2.16). When children were grouped according to sleep efficiency levels, low efficiency sleepers showed a shorter TST (520.9 ± 30.7 min, range: 411.6-576.9) than high efficiency sleepers (535.8 ± 27.7 min, range: 481.7-625.4, *t* = 2.22, *p* = .03, *d* = .51). Low efficiency sleepers also had longer SOL and WASO (SOL: 20.1 ± 11.8, WASO: 37.9 ± 9.8) as compared to the highest efficiency sleepers (SOL: 8.7 ± 1.1, WASO: 29.7 ± 9.0, all *p values* < .001, *d* > .87). Interestingly, low efficiency sleepers went to bed earlier (21:34 ± 00:41) than the highest (21:53 ± 00:40, *t* = 2.0, *p* = .048, *d* = .46) while wake-up time remained no significantly different (*p* = .84).

When comparing the discrepancy between estimations from actigraphy and sleep diary, low efficiency sleepers showed a larger overestimation of their TST compared to children with a high sleep efficiency for all study periods (all days: 107.9 vs 76.5 min, *t*_38_ = 3.4, *p* = .001, *d* = .79; school days: 118.3 vs 88.0 min, *t*_38_ 2.3, *p* = .028, *d* = .53; weekends: 97.4 vs 65.1 min, *t*_38_ = 2.1, *p* = .036, *d* = .51). When school days and weekends were considered separately, the overestimation was even greater during the weekends as compared to school days (see **Table 3**). The discrepancy of WASO measured during school days was significantly different between the groups, indicating a larger underestimation in low efficiency sleepers compared to high efficiency sleepers (31.3 vs 19.6 min, *t*_38_ =-2.9, *p* = .025, *d* = -.54). There was no significant difference between the two groups regarding their bedtime, wake-up time and SOL estimations.

**Table 3:** Mean discrepancy (and standard deviation) between measures (actigraphy estimations minus children or parents’ estimations) for high efficiency and low efficiency sleepers according to the study period.

Discrepancy between actigraphy and sleep diary				Discrepancy between actigraphy and parents’ report			
	High efficiency sleepers (*N = 38)*	Low efficiency sleepers (*N* = 38)	*p*		High efficiency sleepers (*N* = 38)	Low efficiency sleepers (*N* = 38)	*p*
**All days**				**All days**			
Bedtime (min)	40.9 (29.0)	40.0 (29.5)	.89	Bedtime (min)	40.8 (40.7)	32.2 (39.1)	.39
Wake-up time (min)	14.8 (32.8)	17.7 (38.9)	.74	Wake-up time (min)	-9.8 (28.7)	-9.9 (29.3)	.98
TST (min)	-76.5 (39.2)	-107.9 (40.2)	< .001***	TST (min)	-24.0 (42.3)	-50.5 (34.3)	.005**
SOL (min)	4.4 (15.3)	10.1 (23.7)	.24	SOL (min)	NA	NA	NA
WASO (min)	21.2 (24.0)	30.7 (18.5)	.065	WASO (min)	NA	NA	NA
**School days**				**School days**			
Bedtime (min)	29.0 (37.4)	26.5 (34.4)	.77	Bedtime (min)	39.3 (46.8)	28.6 (33.9)	.26
Wake-up time (min)	8.1 (28.0)	8.3 (35.4)	.98	Wake-up time (min)	-1.6 (36.8)	- 6.5 (31.6)	.54
TST (min)	-88.0 (59.4)	-118.3 (54.2)	.028*	TST (min)	-34.1 (57.2)	-53.8 (59.9)	.16
SOL (min)	3.2 (13.9)	6.0 (30.1)	.62	SOL (min)	NA	NA	NA
WASO (min)	19.6 (24.5)	31.3 (18.0)	.025*	WASO (min)	NA	NA	NA
**Weekends**				**Weekends**			
Bedtime (min)	52.9 (45.8)	53.4 (43.2)	.96	Bedtime (min)	42.4 (55.7)	35.7 (59.1)	.62
Wake-up time (min)	21.5 (50.3)	27.1 (60.0)	.67	SOL (min)	-18.8 (37.3)	-13.4 (50.5)	.60
TST (min)	-65.1 (50.0)	-97.4 (74.9)	.036*	WASO (min)	-13.8 (45.7)	-47.2 (35.8)	.001**
SOL (min)	5.6 (19.0)	14.2 (23.4)	.10	SOL (min)	NA	NA	NA
WASO (min)	22.8 (27.3)	30.2 (25.1)	.24	WASO (min)	NA	NA	NA

TST, Total Sleep Time; SOL, Sleep Onset Latency; WASO, Wake After Sleep Onset; NA, Not Available. *p < .05, **p < .01; ***p < .001.

When comparing discrepancy between estimations from actigraphy and parents’ report, all day measures suggested that parents of low efficiency sleepers presented with a larger overestimation of TST than parents of high efficiency sleepers (50.5 vs 24.0 min, *t*_38_ = 2.89, *p* < .005, *d* = .69). This overestimation mainly concerned TST during weekends (47.2 vs 13.8 min, *t*_38_ = 3.40, *p* = .001, *d* = .81), but not during school days (*p* = .16). None of the other comparisons reached statistical significance.

### Agreement Between Measures

#### Actigraphy-Sleep Diary

**Figure 1:**
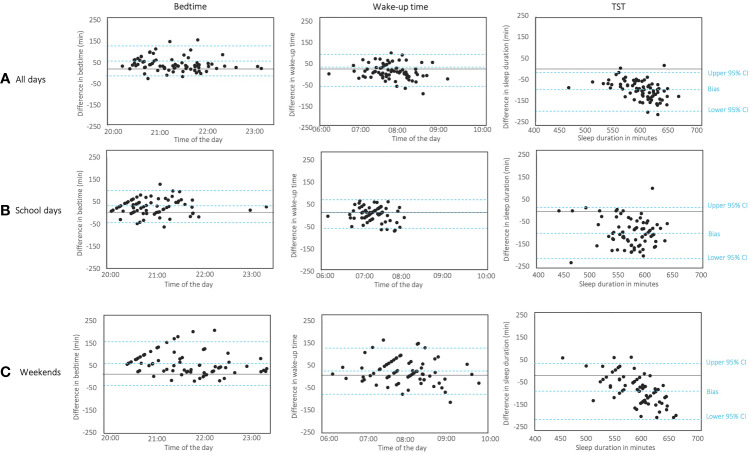
Bland-Altman plots to assess the limits of agreement between actigraphy and sleep diary for bedtime (on the left), wake-up time (in the middle) and Total Sleep Time (TST, on the right) according to **(A)** all days, **(B)** school days, and **(C)** weekends. Each child’s estimation is represented by a dot. Y-axis represents the difference between the two assessed measures being assessed; x-axis represents the average of the two methods. The horizontal line represents the bias; the two horizontal lines representing the 95% limits of agreement, which define the range in which 95% of the differences between methods are expected to fall and are calculated as the bias ± 1.96 standard deviation.

Both bedtime and wake-up time showed low to high significant correlations between the two measures irrespective of the study period (r values between .26 and .77, see **Table 4**). The Bland-Altman plots revealed a satisfactory level of agreement for bedtime and wake-up time between these two measures for all the studied periods. For almost all participants, the difference between actigraphy and sleep diary fell between limits of agreement (see **Figure 1**) and bias remained constant for all bedtime and wake time means. The test of difference in variance did not show significant variability between the two measures (Pitman’s test: all *p*s > .11; see **Table 5**).

**Table 4:** Correlation between sleep parameters assessed by actigraphy, sleep diary, and parents’ report during all days, school days, and weekends.

		All days		School days		Weekends	
		Actigraphy	Sleep diary	Actigraphy	Sleep diary	Actigraphy	Sleep diary
**Bedtime**	**Actigraphy**		*r = *.772*** p < .001		*r = *.586*** p < .001		r = 739*** p < .001
	**Parents’ report**	*r = *.376*** p < .001	*r = *.466*** p < .001	*r = *.350*** p = .002	*r = *.408** p < .001	*r = *.231* p = .046	.262* p = .028
**Wake-up time**	**Actigraphy**		*r = *.447*** p < .001		*r = *.262* p = .027		.448*** p < .001
	**Parents’ report**	*r = *.489*** p < .001	*r = *.507*** p < .001	*r = *.095 *p = .425*	*r = *.096 *p* = .419	*r = *.482*** p < .001	.585*** p < .001
**TST**	**Actigraphy**		*r = *.433*** p < .001		*r = *.105 *p* = .382		.179 *p = .135*
	**Parents’ report**	*r = *.305** p = .008	*r = *.197 *p = .102*	*r = *.119 *p* = .321	*r = *.293* p = .016	*r = *.208 *p* = .075	.188 *p = .120*
**SOL**	**Actigraphy**		*r = *.175 *p = .144*		*r = *.209 *p* = .080		.114 *p* = .340
**WASO**	**Actigraphy**		*r* = -.009 *p = .939*		*r = *.018 *p* = .885		*r = *.011 *p* = .929

TST, Total Sleep Time; SOL, Sleep Onset Latency; WASO, Wake After Sleep Onset. *p < .05, **p < .01; ***p < .001.

**Table 5:** Levels of agreement between the three measures for all sleep parameters during all days, school days, or weekends.

	ACTIGRAPHY vs. SLEEP DIARY				ACTIGRAPHY vs. PARENTS’ REPORT				SLEEP DIARY vs. PARENTS’ REPORT			
	Bias (95% IC)	Limits of agreement Lower; upper	Pitman’s test		Bias (95% IC)	Limits of agreement Lower; upper	Pitman’s test		Bias (95% IC)	Limits of agreement Lower; upper	Pitman’s test	
			Variance *(r*)	*p* value			Variance *(r*)	*p* value			Variance *(r*)	*p* value
**All days**												
Bedtime (hrs:min)	40.5 (33.6; 97.4)	-16.4; 109.6	.11	.37	36.6 (27.4; 45.7)	-41.6; 114.7	.48	< .001***	3.2 (-12.6; 6.1)	-80.6; 74.2	.55	<.001***
Wake-up time (hrs:min)	16.3 (7.8; 24.8)	-53.8; 86.4	.07	.51	-9.7 (-16.4; -2.9)	-67.4; 48.0	.12	.32	-24.9 (-32.6; -17.2)	-88.6; 38.8	.27	.018*
TST (min)	-92.4 (-102.5; -82.4)	-175.7; -9.2	.43	<001***	-36.5 (-45.4; -29.9)	-114.5; 42.1	.21	.04*	56.0 (44.6; 67.4)	-48.4; 158.0	.04	.76
SOL (min)	7.3 (2.6; 12.0)	-32.0; 46.6	.48	<.001***	NA	NA	NA	NA	NA	NA	NA	NA
WASO (min)	26.0 (20.9; 31.2)	-16.7; 68.8	.55	.001***	NA	NA	NA	NA	NA	NA	NA	NA
**School days**												
Bedtime (hrs:min)	27.7 (19.3; 36.2)	-42.1; 97.6	02	.86	34.0 (24.6; 43.5)	-46.3; 114.4	.23	.046*	7.6 (-1.2; 16.5)	-67.2; 82.5	.22	.070
Wake-up time (hrs:min)	8.3 (0.8; 15.8)	-53.9; 70.4	.18	.12	-3.7 (-11.6; 4.2)	-70.5; 63.1	.17	.14	-12.0 (-20.9; -3.2)	-85.2; 61.2	.03	.80
TST (min)	-103.4 (-117.2; -89.6)	-218.0; 11.2	.07	.57	-43.5 (-57.5; -29.6)	-159.0; 71.9	.04	.73	57.8 (45.5; 70.1)	-40.9; 156.5	.02	.81
SOL (min)	4.6 (-0.9; 10.2)	-41.4; 50.6	.41	<.001***	NA	NA	NA	NA	NA	NA	NA	NA
WASO (min)	25.5 (20.3; 30.8)	-17.7; 68.8	.52	<.001***	NA	NA	NA	NA	NA	NA	NA	NA
**Weekends**												
Bedtime (hrs:min)	53.2 (42.7; 63.6)	-33.4; 139.8	.18	.11	39.1 (25.9; 52.2)	-72.8; 151.0	.56	<.001***	-14.1 (-29.2; 1.0)	-138.9; 110.8	.66	<.001***
Wake-up time (hrs:min)	24.3 (11.3; 37.4)	-83.6; 132.3	.17	.13	-16.1 (-26.6; -5.9)	-102.5; 70.3	.36	.024*	-39.6 (-50.5; -28.7)	-128.9; 49.7	.43	<.001***
TST (min)	-81.5 (-96.9; -66.0)	-209.7; 46.8	.63	<.001***	-29.6 (-39.7; -19.5)	-114.8; 55.6	.25	.029^*^	51.4 (35.2; 67.6)	-81.8; 184.6	.46	<.001***
SOL (min)	10.0 (4.8; 13.7)	-32.4; 52.3	.15	.19	NA	NA	NA	NA	NA	NA	NA	NA
WASO (min)	26.6 (20.3; 32.8)	-25.0; 78.1	.47	<.001***	NA	NA	NA	NA	NA	NA	NA	NA

Biases between measures are expressed as actigraphy minus sleep diary, actigraphy minus parents’ report and sleep diary minus parent’s report. Negative values indicate that the first measure overestimated the sleep parameter, whereas a positive value indicate that the first measure underestimated the sleep parameter. Levels of agreement for all comparisons during all days, school days and weekends for each sleep parameter are presented according to Bland-Altman analyses. Variance was determined by the Pitman’s test of difference in variance between the two measures.

TST, Total Sleep Time; SOL, Sleep Onset Latency; WASO, Wake After Sleep Onset; NA, Not Available; p values indicate the significance of Pitman’s test; * p < .05, *** p < .001.

We found a significant correlation between TST reported by actigraphy and sleep diary but only when measures were considered for all days (*r* = .43, *p* < .001). Bland-Altman plots revealed that almost all data were inside agreement limits, but the negative bias tended to increase with the increase of means for all days and weekend plots. Pitman test confirmed a significant difference (both *p*s < .001) in the variability between actigraphy and sleep diary for these two study periods (see **Figures 1A, C**). When measurements concerned the school days, a good agreement with no significant variability between the two measures was found (Pitman’s test *p* = .57), however the limits of agreement (-218; 11.2) and the difference obtained (-103 min) between the two measures were wide (see **Figure 1B**).

Agreement analyses for SOL only showed a satisfactory agreement between actigraphy and sleep diary for the weekend measures (Pitman’s test *p* = .19), without any correlation between the two measures. No correlation or agreement were found for WASO parameters.

#### Actigraphy-Parents’ Report

Significant correlations between the two methods were obtained regarding bedtimes and wake-up times (*r* values between .23 and .49), except for wake-up time during school days (*p* = .43). When all days and school days were considered, a significant agreement between actigraphy and parents’ report was found for wake-up time (Pitman test *p* = .32 and *p* = .14, respectively) but not for weekends measures (*p* = .024). Regarding TST when all days were considered, parents’ reports and actigraphy estimates showed a weak correlation (*r* = .31, *p* = .008) but no agreement (Pitman’s test *p* = .04). A significant agreement was obtained for TST measured during school days; however, the two measures did not correlate (*p* = .12).

#### Sleep Diary—Parents’ Report

Significant correlations between the two methods were found for bedtime (for all study periods, *r* values between .23 and .38, *p* values between .046 and < .001) despite no satisfactory agreement (Pitman’s *p* between .070 and < .001). Wake-up time was significantly correlated between children and parents’ estimations for all days (*r* = .49, *p* < .001) and weekends (*r* = .48, *p* < .001), but a satisfactory agreement was only observed during schooldays (Pitman’s test *p* = .80). Regarding TST, the comparison between the two measures showed significant correlation for all days (*r* = .31, *p* = .008) and satisfactory agreement for all days and school days (Pitman’s test *p* = .76 and *p* = .81 respectively).

## Discussion

This study builds on a vast literature on the validity of subjective sleep measures in pediatrics. We examined sleep parameters through 7-nights actigraphy measures, parents’ reports, and sleep diaries and assessed agreement between these three measures. The originality of our study is that, unlike previous studies in which parents were requested to complete sleep diaries, sleep diaries were herein directly filled in by children.

Several studies exploring sleep of children by asking parents to complete sleep diaries showed an overestimation of sleep duration, inaccurate bedtime and time spent awake during the night compared to objectives measures (15, 21, 23, 33). Overestimation of sleep duration in the literature ranged from 30 to 113 min per night (18, 34) and might be explained by a difficulty to assess sleep latency and night waking in children who become more likely to maintain quiet wakefulness in bed (35, 36). In the present study, we observed a similar level of discrepancy when children completed their own sleep diary. As their parents, children overestimated their sleep duration (92 min) and demonstrated significant difficulties to assess the amount of time they spent awake during the night by overestimating their sleep latency and wake after sleep onset (7 min and 26 min, respectively). One prior study used similar methodology in older children (11 to 12 years) to compare actigraphy and self-reported sleep parameters (20). Authors found that children self-report overestimated TST by 73 min, sleep onset by 21 min and WASO by 50 min. Several factors might explain such discrepancy between measures. As suggested by Lockley, Skene, and Arendt (37), even though these two methods attempt to measure sleep, they may measure different aspects of sleep since sleep diary relies on a subjective recollection of sleep, and actigraphy reports motor activity. Moreover, it is noteworthy that actigraphy as an objective measurement also has limitations, in particular with an overestimation of wake during sleep period (28, 38, 39) and sleep latency (40) when compared to the gold standard PSG recording.

In the present study, parents provided better estimation of sleep duration (+36 min) than children (+ 92 min) compared to actigraphy. When comparing self-report, parents’ reports and PSG, Combs and colleagues (23) found that parents and children overestimated TST, sleep efficiency, and sleep latency compared to PSG. In contrast with our results, the differences between children and parents were less than 5 min and children provided a better estimation of TST than their parents. In this study children were older (age 9 to 17 years), and closer to those recorded by Short and colleagues (17) who previously showed that between 13 to 17 years old, adolescents’ reports were more accurate than parents’ reports. These results may suggest that sleep perception become more accurate with maturation.

On the basis of Bland-Altman tests, we observed that parents’ report did not display a satisfactory agreement with actigraphy as in other studies (15, 18, 34). Only parents’ perceptions of sleep duration and wake-up time were associated with sleep parameters as derived from actigraphy. However, actigraphy and sleep diaries completed by children regarding bedtime and wake-up time showed good agreement. The estimation of sleep duration during school days also showed satisfactory agreement, albeit to a lesser extent regarding the wide individual differences between the two measures. The current study further suggests that no agreement between actigraphy and children diaries was obtained for sleep onset latency or wake after sleep onset. Similar findings have been reported by Werner and colleagues (28) when actigraphy data were compared to diaries filled by parents. They found a satisfactory agreement regarding sleep start, sleep end, and sleep period, but not for nocturnal sleep and wake time. In accordance with previous studies, our results suggested that sleep diary completed by children or parents’ report provide interesting measures of self-perception. However, because of their insufficient agreement for sleep duration and nocturnal wake time, actigraphy is a more appropriate choice when clinical or research assessment need accurate estimate of children’s sleep.

Our results also suggested that school days and weekends should be analyzed separately. Children and parents’ accuracy to estimate sleep parameters was different during these two periods. Total sleep time overestimation was greater for school days than weekends when children or parents’ reports were compared to actigraphy. While actigraphy objectively reported a classical reduction of sleep duration during school days compared to weekends, parents and children estimated that sleep duration was similar between these two periods. As previously reported, when sleep parameters are considered for the entire week, parents tend to report earlier bedtimes and later wake-up times in comparison with actigraphic measures (17, 18). However, we found that wake-up time was better estimated by parents during school days than weekends. This result may be explained by a greater involvement in waking their child during school days compared to weekends, which allow them to be able to provide accurate wake time estimates. These results highlighted that sleep assessments lasting for a week are clinically important to apprehend sleep variation from school days to weekends, particularly if children “oversleep” on weekends to compensate for a lack of sleep (17, 41, 42). Averaging data from weekdays and weekends might wipe out those variation. Acebo and colleagues (26) and Short and colleagues (43) respectively suggested that actigraphy and sleep diary need at least five nights of recording to provide adequate stability for sleep parameters. Considering weekends assessment, two nights of sleep diaries have been shown to be insufficient to provide reliable estimation (43).

A number of factors may influence the measurement or perception of sleep parameters. Sleep quality itself has been shown to influence the congruence between subjective and objective measures (10). We found that discrepancy between objective and subjective measures was greater for children with lower sleep efficiency (< 89%). In this group, sleep time reported both by children in sleep diary and by their parents was overestimated in greater extent (> 30 min) compared to children with higher sleep efficiency. These children also underestimated their time spent awake during the night compared to those with higher sleep efficiency. Our results are in accordance with those reported by Van Den Berg and colleagues (44) in a cohort of elderly persons showing that subjects with poor sleep quality as measured by actigraphy consistently overestimated their sleep duration. Actigraphic measures of poor sleep quality such as shorter TST, lower sleep efficiency and longer SOL, were all associated with a higher diary estimates of TST than actigraphic measures. An earlier bedtime, later wake-up time, poor cognitive function and male gender were also associated with a higher level of disagreement. In children, inaccurate estimation of sleep indices might also arise from cognitive factors, such as the general level of cognitive functioning, the capacity to estimate time, the motivation to recall sleep parameters, or the ability to maintain in long term memory such information. Sleep diary filled by children associated with actigraphy could be an interesting tool to evaluate parameters that could contribute to adjust subjective perception to objective sleep values.

Our ability to define the study as a school project and the perfect consent rate we obtained (100%) discard consent or selection biases. Because feasibility of the sleep diary within the home environment and within the family schedules might affect compliance and increase variability between children (45), we have chosen to have sleep diaries filled-in upon the arrival at school, under teachers’ supervision. Teachers’ involvement every morning during this task might have contributed to improve data collection. However, we cannot rule out that results would have been the same if sleep diaries were completed at home.

This study presents limitations that need to be considered in interpreting the results. First, we included a non-clinical cohort of children for most of them middle-class Caucasian children, which may not allow findings to generalize to children suffering from sleep disorders or to samples characterized by greater demographic and socio-economic heterogeneity. Additionally, as mentioned two nights of weekend sleep diary entries may be insufficient to estimate valuable sleep indices because of the larger variance in weekend sleep patterns (43). Finally, the present study investigated the degree of convergence between subjective measures and actigraphy which is known to present some limitation to estimate wake during sleep period. Subjective sleep assessments should be compared to the gold standard polysomnography in longitudinal studies to provide information about sleep perception evolution during life span.

## Conclusion

The comparison between objective sleep measures, children self-reports and parents’ report is useful to depict perception of children’s sleep. The present study suggests that children aged between 8 and 9 are mature enough to complete a 7-day self-reporting of their sleep. Despite the classical indices of misperception, we found a good level of agreement between sleep-diary and actigraphy for bedtime and wake-up time, as for the total sleep time obtained during school days. Our results support the importance to analyze separately school days and weekends when we record sleep in children. Despite, the discrepancy found between subjective and objective measurements, the results of this study call for research on how the idealized parents and children perception of their sleep could be adjusted. Sleep diary associated with actigraphy could be an interesting tool to evaluate parameters that could contribute to reset subjective perception to objective sleep values.

## Data Availability Statement

The datasets generated for this study are available on request to the corresponding author.

## Ethics Statement

The studies involving human participants were reviewed and approved by Comité d’Ethique pour les Recherches Non Interventionnelles, Grenoble, France. Written informed consent to participate in this study was provided by the participants’ legal guardian.

## Author Contributions

SM and AR developed the study concept. All authors contributed to the study design. Testing and data collection were performed by SM and AR. SM, HB, and AR performed the data analysis and interpretation. SM and AR drafted the manuscript. HB provided critical revisions. All authors approved the final version of the manuscript for submission.

## Funding

French National Agency for Research (ANR, grant n°ANR-15-CE33-0003) and Prevent’Horizon chair.

## Conflict of Interest

The authors declare that the research was conducted in the absence of any commercial or financial relationships that could be construed as a potential conflict of interest.
